# Rennet-Induced Mixed Yak–Cow Casein Micelle Gels: Gelation Properties and Formation Mechanisms

**DOI:** 10.3390/gels12080683

**Published:** 2026-08-03

**Authors:** Puwei Yan, Shaobo Zhen, Liya Zhang, Zhaobin Guo, Yan Zhang

**Affiliations:** 1 College of Food Science and Engineering, Gansu Agricultural University, Lanzhou 730070, China; yanpw@st.gsau.edu.cn (P.Y.);; 2 School of Hotel Management, China University of Labor Relations, Beijing 100089, China

**Keywords:** yak milk, casein micelles, rennet-induced coagulation

## Abstract

Rennet-induced yak casein gels have longer gelation time but higher elastic modulus than bovine casein gels, which limits cheese processing efficiency. In this study, yak and bovine casein micelles were mixed at ratios of 100:0, 75:25, 50:50, 25:75, and 0:100 (denoted as Y100, Y75, Y50, Y25, and Y0, respectively). HPLC was used to monitor the relative peak area of released glycomacropeptide (GMP) during κ-casein hydrolysis (normalized to the 60-min maximum) to characterize rennet catalysis kinetics, and the effects on rheology, texture, and microstructure were analyzed. The results showed that the mixing ratio significantly modulated the hydrolysis rate of κ-casein, storage modulus (G′), hardness, chewiness, adhesiveness, and gel network compactness. Among them, Y75 (75% yak casein) exhibited the best performance, with G′ reaching 1709.78 Pa and a gel induction time of only 11.67 min; its hardness, chewiness, and adhesiveness were 1.46, 1.22, and 1.59 times those of pure bovine casein gel, respectively, and its microstructure showed a more uniform and dense pore distribution. In conclusion, optimizing the yak–bovine casein micelle ratio (75:25) can significantly improve gel quality while maintaining processing efficiency, providing a theoretical basis for mixed-milk cheese production, though further validation in real whole-fat milk systems is required.

## 1. Introduction

Yak is a livestock species adapted to high-altitude (2500–6000 m), cold environments and is distributed across the Qinghai–Tibet Plateau of China, with this population accounting for more than 95% of the global yak population [1]. For centuries, yaks have provided essential food and income for local pastoralist communities. The annual production of yak milk in China is estimated at approximately 1.2 million tons [2].

Yak milk is characterized by a high solids content, containing 16.9–17.7% dry matter, 4.9–5.3% protein, and 5.5–7.2% fat [3], making it particularly suitable for cheese manufacturing. Previous studies have demonstrated its superior cheese-making properties. The cheese yield from yak milk is approximately 1.67-fold higher than that from cow milk, and yak Cheddar cheese exhibits greater hardness during ripening. Despite these advantages, most yak milk is consumed as fresh milk or processed into traditional dairy products, i.e., Qula and buttered tea, with only a small proportion utilized for high-value-added products [4]. In recent years, however, more than one-third of yak milk production has been directed toward cheese manufacturing [5]. Within China’s cheese market, fresh curd mozzarella is the largest single category, accounting for more than 50% of the total consumption [6]. The development of high-value-added yak cheese products therefore has substantial potential to promote economic growth in less developed yak-producing regions.

Rennet-induced milk coagulation is a key step in cheese manufacture and is closely associated with both cheese quality and economic efficiency [7]. Numerous studies have shown that the structure and physicochemical properties of casein micelles [8], such as their average size, micellar calcium concentration [9], and relative κ-casein content [10], are key determinants of rennet coagulation behavior, including gelation time and gel strength.

Yak casein micelles are larger, with a Z-average (intensity-weighted mean hydrodynamic diameter determined by dynamic light scattering at 25 °C) of 218.6 nm, and contain a lower relative proportion of κ-casein (~13%) but a higher concentration of total calcium than cow casein micelles. As a result, yak milk forms a more compact and firmer rennet-induced gel network, exhibiting a higher elastic modulus (G’ = 6.5 Pa) compared with cow milk (G’ = 2.5 Pa). However, yak casein micelles require a longer gelation time (11.6 min) than cow casein micelles (8.7 min) [11].

Despite these findings, the structure and physicochemical properties of yak casein micelles, particularly their internal organization and intramolecular interactions, remain insufficiently characterized. Furthermore, the mechanisms by which these micellar properties influence rennet-induced coagulation require more systematic investigation.

Therefore, it is essential to investigate the coagulation behavior, textural properties, and microstructure of rennet-induced mixed yak and cow casein gels. Casein micelles from yak and cow were isolated using membrane separation techniques and used as raw materials to prepare systems containing different yak-to-cow casein micelle ratios. The physicochemical properties, aggregation kinetics, and network microstructure of rennet-induced mixed yak–cow casein micelle gels were systematically evaluated. This study provides a novel strategy for the high-value utilization of yak milk.

## 2. Results and Discussion

### 2.1. Particle Size of Yak–Cow Casein Micelle Complexes

**Figure 1:**
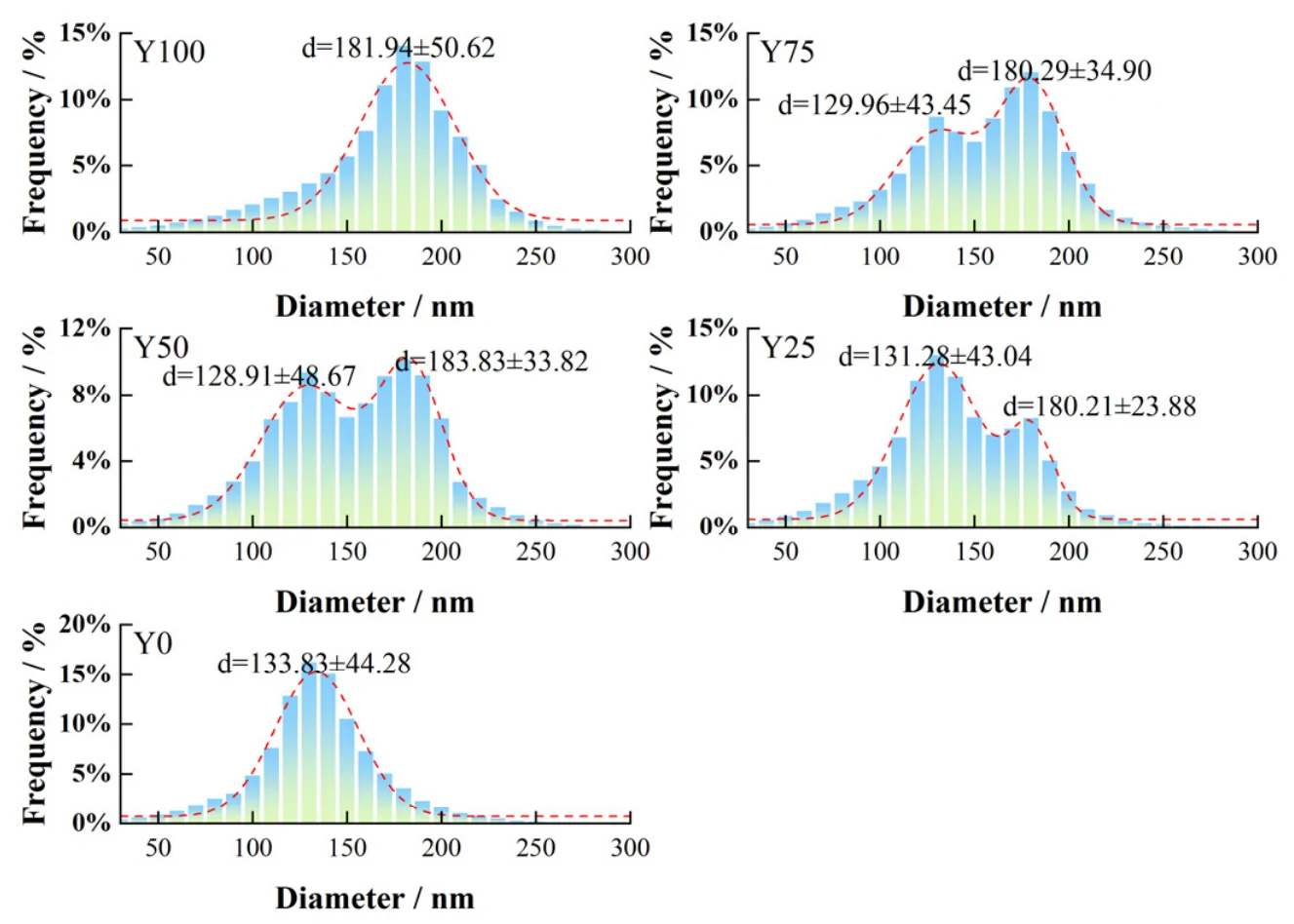
Average particle size of yak–cow casein micelle complexes. The colored bars represent the frequency of particle size distribution, and the red dashed line indicates the corresponding Gaussian fitting curve. Data are presented as mean ± SD.

The particle size distributions of yak–cow casein micelle complexes at different blending ratios are shown in Figure 1. Cow casein micelles (Y0) exhibited a monomodal distribution with an average particle diameter of 133.83 ± 44.28 nm, while yak casein micelles (Y100) also exhibited a monomodal distribution but with a significantly larger mean diameter of 181.94 ± 50.62 nm, which is consistent with the findings of Zhang [11]. This result further confirms that the structural integrity of casein micelles was maintained during membrane separation. Upon the addition of cow casein micelles, notable changes in the particle size distribution were observed. Y75, Y50, and Y25 displayed bimodal distributions, characterized by a secondary shoulder peak at ~130 nm, while the main peak remained near 180 nm. This result indicates good dispersion of both types of micelles without significant aggregation before rennet addition, a result consistent with the study of Bulca [12].

### 2.2. Glycomacropeptide (GMP) Release of Mixed Casein Micelle Solutions After Rennet Addition

**Figure 2:**
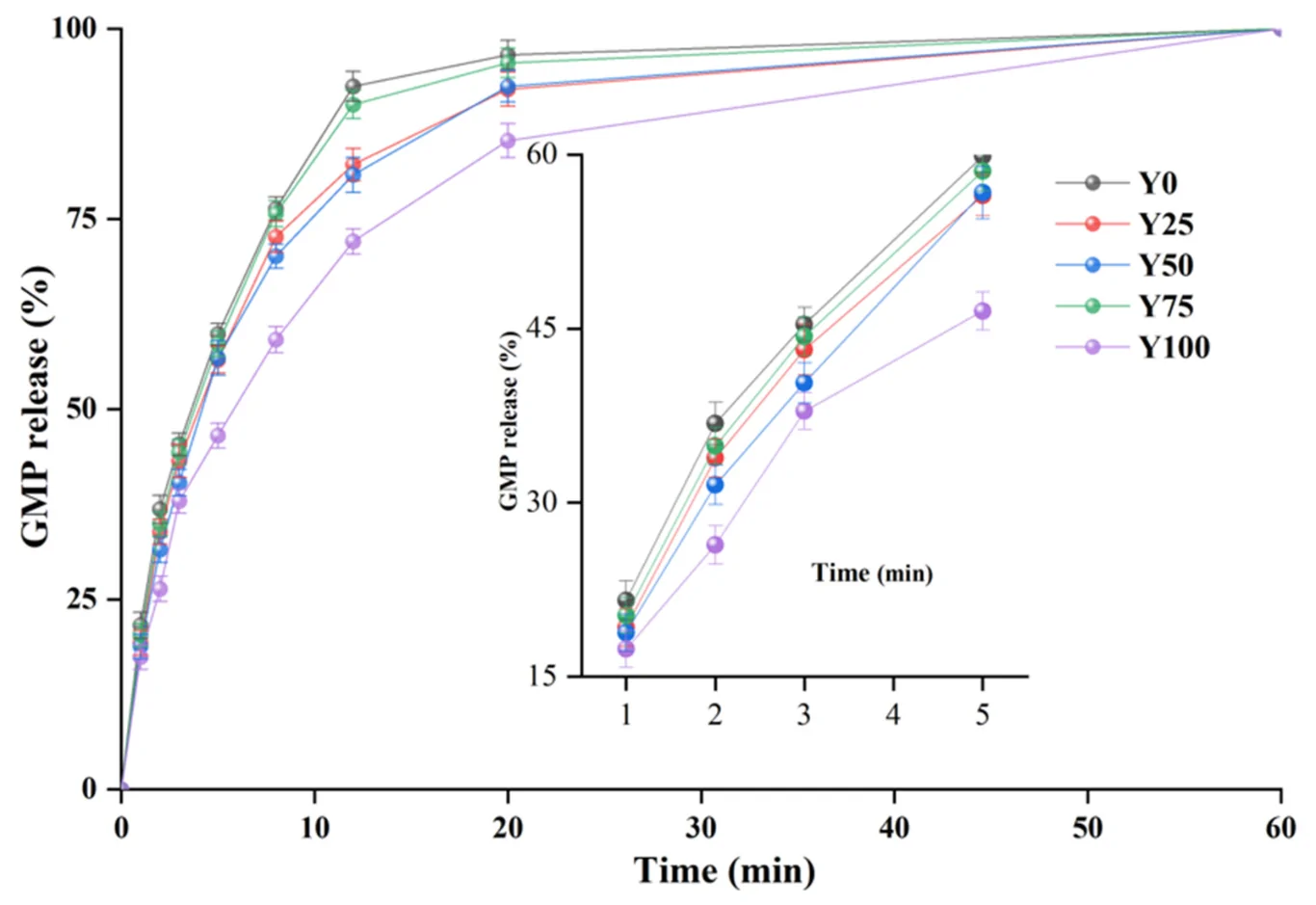
GMP release kinetics of yak–cow casein micelle complex.

The kinetics of GMP release from mixed yak–cow casein micelle solutions following rennet addition are shown in Figure 2. GMP release at each time point was normalized to the maximum level observed at 60 min. Across all formulations, GMP release increased progressively over time and followed a characteristic enzymatic reaction profile. The hydrolysis process included an initial rapid release phase, followed by a relatively stable linear growth phase, and ultimately reached a plateau due to substrate depletion. These findings are consistent with results reported by Ono [13]. During the initial stage, rennet cleaves κ-casein at the Phe105-Met106 bond located on the micelle surface, resulting in a sharp increase in GMP release. Although chymosin specifically cleaves the conserved Phe105–Met106 bond of κ-casein, differences in enzyme accessibility associated with micellar organization may also contribute to the observed hydrolysis behavior [14].

Interestingly, the slope representing the GMP release rate of Y75 (a relative rate based on normalized data) was comparable to that of Y0 and higher than that observed for Y25, Y50, and Y100. In general, yak casein micelles exhibit a lower GMP release rate than cow casein micelles due to their lower κ-casein content [15], which limits rennet cleavage sites and delays reaction initiation [11]. In contrast, the efficient hydrolysis observed in Y75 suggests that the optimized coexistence of yak and cow casein micelles preserves sufficient substrate-accessible κ-casein. This configuration maintains effective enzymatic cleavage kinetics, ensuring that rennet-induced gelation is not kinetically constrained despite the high proportion of yak casein micelles. Furthermore, although cow casein generally contains a higher proportion of κ-casein, the hydrolysis efficiency depends not only on κ-casein abundance but also on micellar organization, enzyme accessibility, and the spatial distribution of κ-casein on the micelle surface [16]. Therefore, the superior GMP release observed in Y75 likely results from the combined effects of composition and micellar structure rather than κ-casein content alone.

### 2.3. Coagulation Properties of Mixed Casein Micelle Solutions

**Figure 3:**
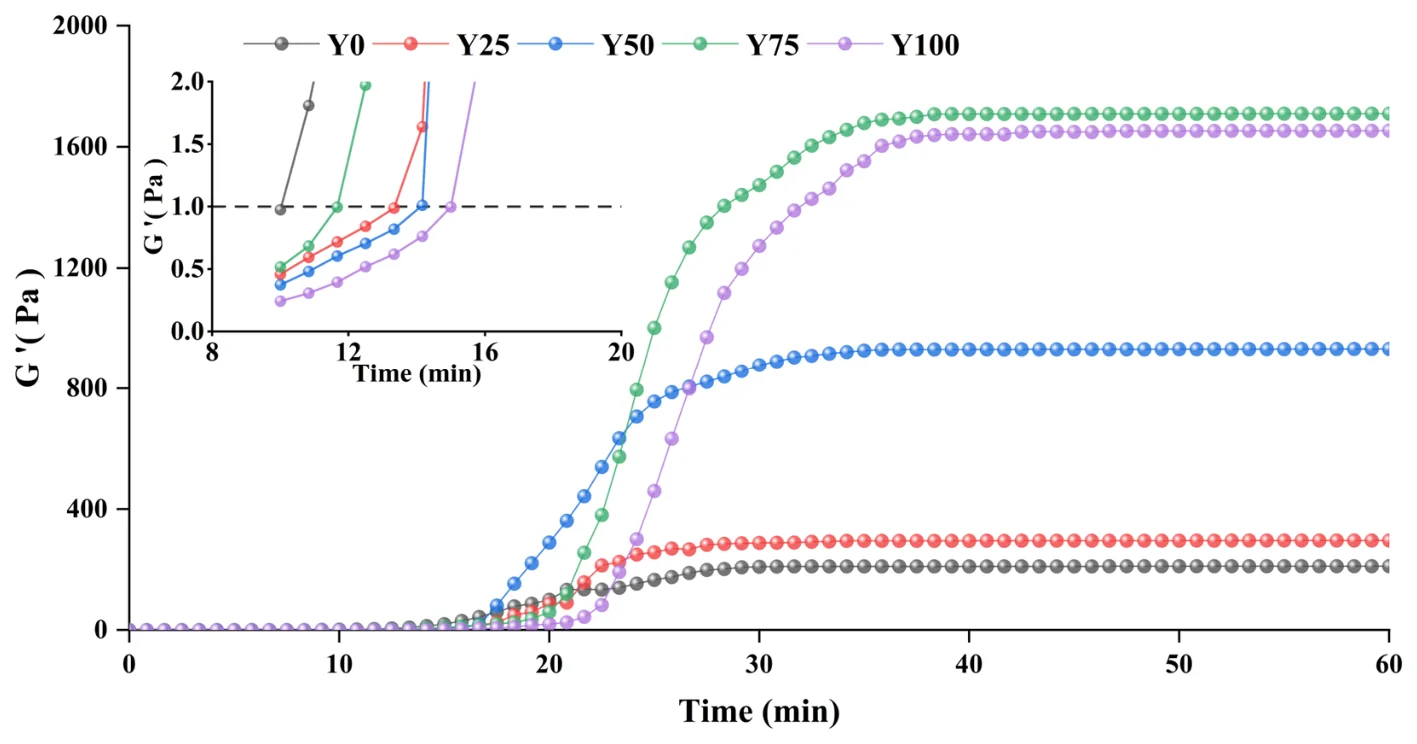
Storage modulus (G’) as a function of time during rennet-induced coagulation of yak–cow casein micelles.

The evolution of storage modulus (G’) of mixed yak–cow casein micelle solutions following rennet addition is shown in Figure 3. Across all formulations, the gelation process exhibited the characteristic stages of rennet-induced coagulation, including an initial lag phase, a rapid modulus increase phase, and a final plateau phase. The blending ratio of yak and cow casein micelles significantly affected the duration and magnitude of these gelation stages (Figure 3). The onset of gelation was defined as the time required for G’ to reach 1 Pa after rennet addition [17]. As shown in Figure 3, Y0 required the shortest gelation time (~10.00 min), which may be due to the smaller micellar size and higher availability of most enzymatic cleavage sites. Y75 showed a shorter gelation time (~11.67 min) than Y25 (~13.33 min), Y50 (~14.17 min), and Y100 (~15.00 min). This result is consistent with GMP release kinetics (Figure 2), indicating that systems with faster initial GMP release exhibit more rapid micelle aggregation.

Notably, Y75 exhibited the highest G’, exceeding even that of gels formed from yak casein micelles alone (Y100, ~1653 Pa). This enhanced rigidity reflects a synergistic reinforcement arising from complementary structural and chemical contributions. Previous studies on mixed casein systems have generally reported that incorporating cow milk into yak milk improves gel properties primarily through enhanced micellar interactions and optimized network organization, whereas the gel strength typically remains lower than or comparable to that of pure yak casein gels [18]. In contrast, the present study demonstrates that an appropriate mixing ratio (Y75) generates a gel network with rigidity exceeding that of pure yak casein, suggesting a genuine synergistic effect rather than a simple intermediate response between the two individual casein systems. From a structural perspective, an interstitial filling effect occurs whereby smaller cow casein micelles occupy and reinforce the void spaces within the coarser yak casein micelle network, creating a denser architecture. From a chemical perspective, the elevated content of colloidal calcium phosphate (CCP) associated with yak casein micelles promotes calcium-mediated cross-linking. As reported by Lucey [19], CCP content is a critical factor positively correlated with gel firmness. Meanwhile, a lower κ-casein content may reduce steric stabilization while simultaneously decreasing the number of available enzymatic cleavage sites [20]. Consequently, gelation time is determined by the balance between enzymatic hydrolysis, micellar destabilization, calcium-mediated aggregation, and network formation.

### 2.4. Intermolecular Interactions in Mixed Yak–Cow Casein Micelle Gels

**Figure 4:**
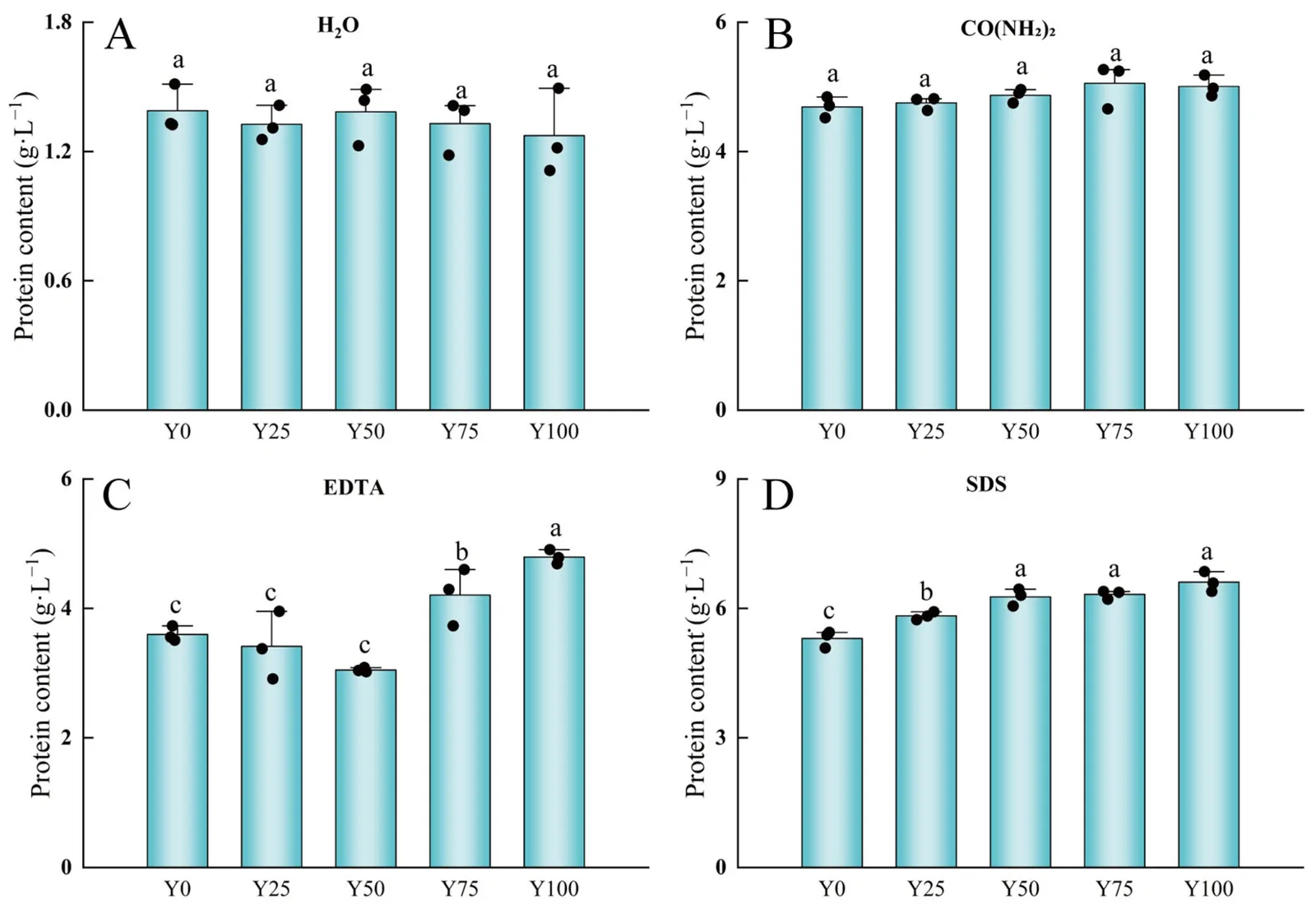
Protein stabilization by individual binding interactions in yak–cow casein micelle gels subjected to different dissociating agents: (**A**) H_2_O, (**B**) CO(NH_2_)_2_, (**C**) EDTA, and (**D**) SDS. Different lowercase letters indicate significant differences (*p* < 0.05).

The stability of rennet-induced casein networks arises from a complex balance of intermolecular forces, including electrostatic interactions, hydrophobic interactions, hydrogen bonds, and calcium-mediated cross-links [21]. To delineate the relative contributions of these interactions, the gels were treated with four dissociating agents (Figure 4). As detailed by Gonçalves [22], sodium dodecyl sulfate (SDS) disrupts hydrophobic interactions, ethylenediaminetetraacetic acid (EDTA) chelates calcium and thereby breaks ionic bridges, and urea (CO(NH_2_)_2_) destabilizes hydrogen bonds, while deionized water serves as a control for unbound proteins. The protein concentration measured in the supernatant varied significantly depending on both the dissociating agent and the gel formulation. In the water-treated control (Figure 4A), a baseline protein release of approximately 1.34 g·L^−1^ was observed. No significant differences were detected among the samples (*p* > 0.05), indicating that deionized water primarily removes weakly associated proteins rather than those integrated in the gel network, a finding consistent with the results reported by Gonçalves [22].

Upon treatment with urea (Figure 4B), the protein concentration in the supernatant increased sharply to approximately 4.88 g·L^−1^. Importantly, no significant differences were observed among the five formulations (Y0–Y100). This suggests that hydrogen bonding acts as a fundamental, ubiquitous stabilizing force within the casein gel network, contributing comparatively to gel integrity regardless of the yak-to-cow casein ratio. This observation is consistent with previous reports demonstrating the universal role of hydrogen bonds in maintaining the structural framework of casein gels [21].

In contrast, treatments with EDTA and SDS revealed distinct species-dependent effects. Upon EDTA treatment (Figure 4C), the solubilized protein content increased significantly with a higher proportion of yak casein micelles. While Y0, Y25, and Y50 exhibited relatively low protein release, significantly higher values were observed for Y75 (3.87 g·L^−1^) and Y100 (4.13 g·L^−1^). Because EDTA selectively chelates calcium ions, this trend confirms that ionic bridging plays a substantial role in stabilizing gels enriched with yak casein micelles. This finding is consistent with previous studies showing that calcium chelation markedly weakens rennet-induced casein gels by disrupting calcium bridges [23]. However, the greater protein release observed in Y75 and Y100 suggests that yak casein-enriched gels are more dependent on calcium-mediated stabilization than gels formed predominantly from cow casein micelles. The observed gel strengthening may be associated with enhanced calcium-mediated interactions, which promote the formation of a denser network of calcium-mediated interactions, although CCP was not directly quantified. Similarly, in the presence of SDS (Figure 4D), protein release increased with increasing proportions of yak casein, with Y50, Y75, and Y100 exhibiting the highest solubilized protein concentrations (~6.39 g·L^−1^). As SDS disrupts hydrophobic interactions, these results suggest that gels containing higher levels of yak casein rely more strongly on hydrophobic associations for network stabilization. Previous studies have recognized hydrophobic interactions as an essential contributor to the structural integrity of rennet-induced casein gels [24]. Whereas the pronounced SDS sensitivity observed in Y75 and Y100 indicates that the contribution of hydrophobic associations becomes increasingly important with increasing yak casein content. Studies have shown that extensive hydrophobic regions make a substantial contribution to the mechanical strength of casein-based gels [25]. In conclusion, while hydrogen bonds provide a baseline structural stability across all formulations, the enhanced mechanical properties of optimized mixed gels (especially Y75 and Y100) are primarily driven by strengthened calcium-mediated cross-linking and hydrophobic interactions associated with the yak casein component.

### 2.5. Microstructural Properties of Rennet-Induced Mixed Yak–Cow Casein Micelle Gels

**Figure 5:**
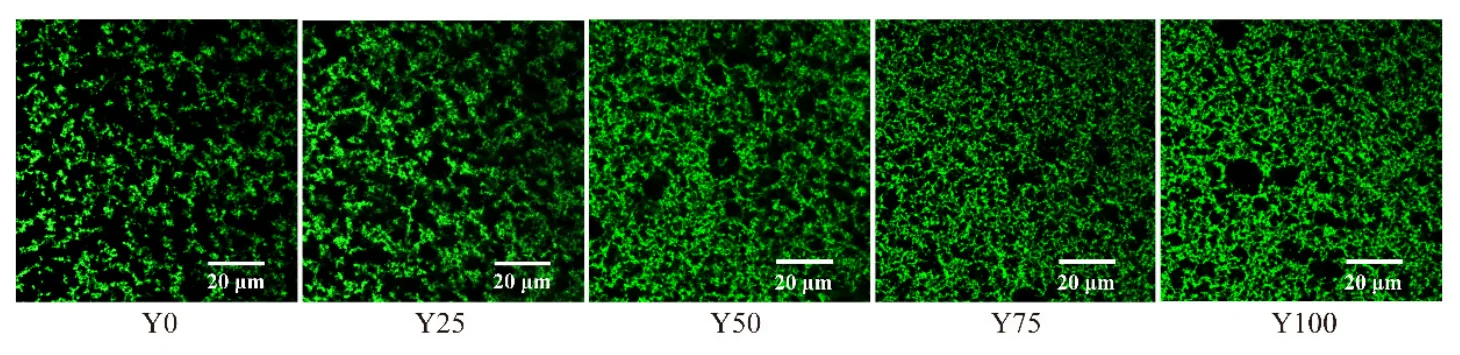
CLSM micrographs of mixed yak–cow casein micelle gels. Scale bar = 20 μm.

The confocal laser scanning microscope (CLSM) observations revealed that all rennet-induced casein micelle gels exhibited a three-dimensional network structure characteristic of protein-based gels (Figure 5). Gels formed from cow casein micelles alone (Y0) displayed a relatively loose and discontinuous particulate network, characterized by large, irregular interstitial voids, and weak fluorescence intensity. This morphology is consistent with previous reports describing cow casein gels as particulate networks with relatively large pores and limited connectivity, which generally correspond to lower gel firmness and elasticity [26]. With increasing incorporation of yak casein micelles, the gel microstructure became progressively more interconnected and compact. Notably, the Y75 system exhibited the densest and most homogeneous microstructure, characterized by the strongest fluorescence signal and the smallest, uniformly distributed pores. The structural optimization in Y75 can be primarily attributed to a synergistic interstitial filling effect, whereby smaller cow casein micelles occupy void spaces between the larger, clustered yak casein micelles, thereby enhancing overall packing density of the protein matrix. Previous studies have demonstrated that incorporating cow casein into yak casein systems can improve gel microstructure by optimizing micellar organization and network connectivity [27]. The exceptionally uniform and compact network observed in Y75 indicates that an optimized mixing ratio produces a stronger synergistic effect than would be expected from simple compositional averaging. Furthermore, the high mineral content associated with yak casein micelles likely facilitated extensive calcium-mediated cross-linking and hydrophobic interactions, which function as molecular connectors that reinforce the gel network [21]. In contrast, gels composed exclusively of yak casein micelles (Y100), while dense, exhibited a coarser structure with larger aggregates. This suggests that, in the absence of cow casein micelles, large yak casein micelles tend to form excessive self-aggregates. These findings are consistent with previous studies on mixed-species milk systems, which have shown that optimized micellar blending ratios minimize network porosity and enhance gel hardness relative to single-milk systems [28].

**Figure 6:**
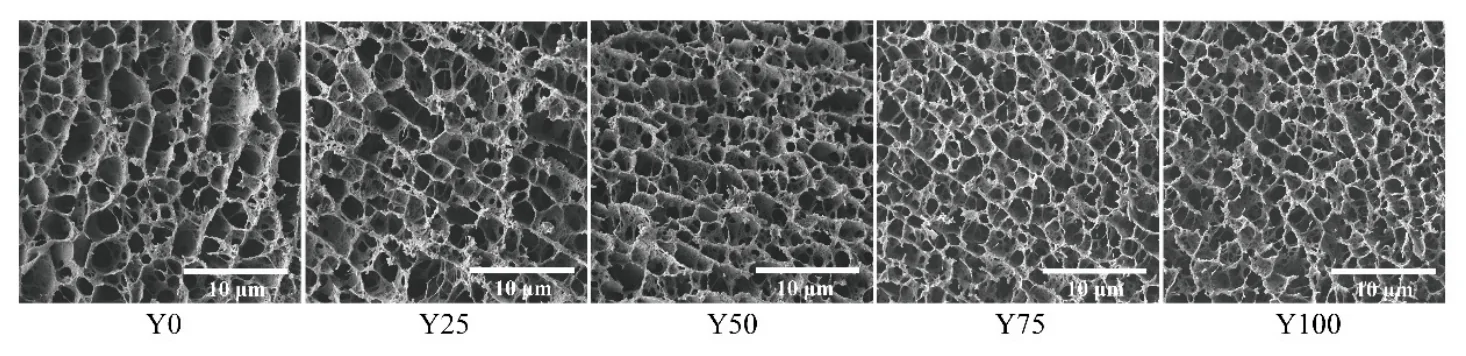
SEM micrographs of rennet-induced yak–cow casein micelle gels. Scale bar = 10 μm.

The scanning electron microscope (SEM) revealed significant microstructural differences among gels formed from yak and cow casein micelles at different blending ratios (Figure 6). Consistent with the CLSM results, Y0 exhibited a highly porous and loose microstructure characterized by extensive interstitial voids, in agreement with the results of Sun [29]. With increasing incorporation of yak casein micelles, the gel network became progressively denser and more compact. Notably, Y75 displayed the most homogeneous architecture, in which protein aggregates appeared to be fused into a continuous, sheet-like matrix with a markedly reduced pore size. Even though Y100 also exhibited a dense structure, its surface morphology was comparatively rougher and more heterogeneous, with irregular aggregates and less structural continuity than those observed in Y75. The superior μ-compactness and uniformity of the Y75 microstructure may facilitate an interstitial filling effect, whereby smaller cow casein micelles occupy void spaces within the yak casein network, reinforced by calcium-mediated cross-linking.

### 2.6. Texture Profile Analysis (TPA) of Rennet-Induced Mixed Yak–Cow Casein Micelle Gels

Textural properties are critical indicators of sensory quality and consumer acceptance of food products. As shown in Table 1, texture profile analysis (TPA) was used to systematically evaluate key rheological parameters of rennet-induced yak–cow casein micelles, including hardness, chewiness and gumminess. The textural parameters exhibited a distinct non-monotonic dependence on the proportion of yak casein micelles.

In general, the addition of yak casein micelles significantly reinforced the gel network, with most mechanical attributes reaching their optimal values at the Y75 ratio. Specifically, hardness increased progressively from 76.20 g in the cow casein micelles alone (Y0) to a maximum of 111.63 g for Y75, corresponding to a 1.46-fold enhancement. Even though gels prepared solely from yak casein micelles (Y100) also exhibited high hardness (106.78 g), this value was slightly lower than that observed for Y75.

A similar synergistic trend was observed for chewiness and gumminess. The Y75 gel exhibited the highest chewiness and gumminess, which were 1.22-fold and 1.59-fold higher, respectively, than those of the Y0 gel. This textural optimization correlates with the denser and more homogenous microstructure observed in CLSM and SEM analyses. The superior hardness and elasticity of Y75 can be attributed to a synergistic packing effect, in which smaller cow casein micelles reinforce interstitial spaces within the yak casein matrix, coupled with enhanced calcium-mediated cross-linking associated with mineral-rich yak casein micelles [30]. Previous studies have demonstrated that improvements in gel texture are closely associated with the formation of a homogeneous and interconnected protein network [31]. The present findings further suggest that optimizing the yak-to-cow casein ratio generates a synergistic structural reinforcement that cannot be achieved by simply increasing the proportion of yak casein.

In contrast, the slight reduction in mechanical strength observed for Y100, despite its high protein and mineral content, likely results from excessive self-aggregation of yak micelles in the absence of smaller cow micelles to regulate spatial organization. These findings confirm that an optimized blending ratio yields a mechanically superior gel network compared with single-species systems.

### 2.7. Mechanism of Rennet-Induced Gel Formation in Mixed Yak–Cow Casein Systems

**Figure 7:**
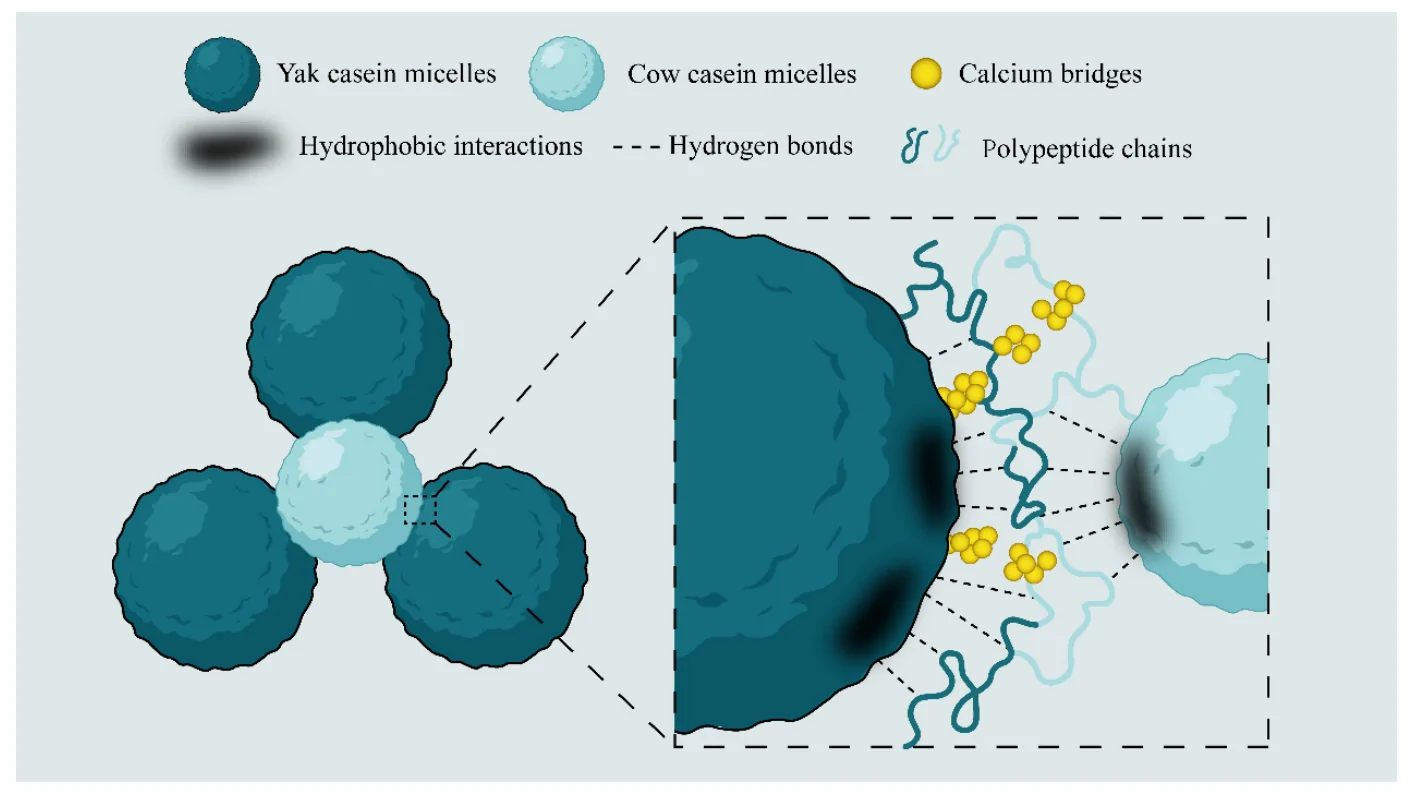
Schematic illustration of the proposed gelation mechanism of the Y75 yak–cow casein micelle co-coagulation system.

Based on the combined physicochemical characterization and microstructural observations, a mechanistic model for rennet-induced gel formation in mixed yak–cow casein micelle systems is proposed (Figure 7). From a structural perspective, gel assembly is driven by a synergistic interstitial filling effect. The larger yak casein micelles form the primary load-bearing framework of the gel network, while the smaller cow casein micelles occupy interstitial spaces between these aggregates. This size-complementary packing increases network compactness, reduces porosity, and promotes spatial continuity of the protein matrix.

At the molecular level, the composite network is stabilized by multiple intermolecular interactions. The high concentration of CCP in yak casein micelles facilitates the formation of extensive calcium bridges (yellow spheres), effectively bridging adjacent micelles and reinforcing network connectivity. In parallel, hydrophobic interactions (dark shadows) between exposed non-polar protein domains contribute to cohesive aggregation following κ-casein hydrolysis, while hydrogen bonding (dashed lines) provides additional stabilization between neighboring protein residues.

The combined effects of dense micellar packing and chemically reinforced cross-linking account for macroscopic stiffness, viscoelastic behavior, and structural integrity observed in the optimized mixed system (Y75).

## 3. Conclusions

Overall, this study provides mechanistic insights into the rennet-induced gelation behavior of mixed yak–cow casein micelle systems and identifies the Y75 formulation as exhibiting the most favorable gel properties among the tested ratios. These findings provide a theoretical basis for optimizing mixed yak–cow milk formulations and may facilitate the development of high-quality yak milk cheese. Nevertheless, because the present work was performed using isolated casein micelle systems, further validation in whole milk and pilot-scale cheese manufacture is required before industrial application.

## 4. Materials and Methods

### 4.1. Yak Milk Collection and Casein Micelle Solution Preparation

Yak milk samples were collected in August from the experimental base in Zhuaxixiulong Town, Gansu Province, China, and transported to the laboratory within 4 h under refrigerated conditions (<4 °C). Upon arrival, sodium azide was added to the milk at a final concentration of 0.02% (*w*/*v*). The milk was centrifuged twice at 6000× *g* for 30 min at 20 °C, followed by filtration through Whatman filter paper. Cow milk samples were concurrently collected from a local experimental base at Gansu Agricultural University and subjected to the same pretreatment procedures. Before blending, the protein concentration of yak and cow casein micelle suspensions was individually standardized. After mixing at the designated ratios, all formulations were adjusted to the same final protein concentration to ensure that differences in gelation behavior were attributable solely to the blending ratio rather than variations in protein concentration.

Casein micelle concentrate solutions were prepared from skim milk using a pressure-driven cross-flow filtration system equipped with an ultrafiltration membrane module (F210-1812C, 50 kDa; Hefei Woteng Membrane Separation Equipment Co., Ltd., Hefei, Anhui, China), following the method of Puri [32]. Ultrafiltration was performed at a transmembrane pressure of 0.14 MPa and a temperature of 25 °C.

The resulting casein micelle concentrates were diluted using simulated milk ultrafiltrate (SMUF) to a final protein concentration of 4.5 g/100 mL, as determined by the Kjeldahl method. SMUF was prepared according to previously reported methods [33], with the following composition (g·L^−1^): 1.58 KH_2_PO_4_, 1.20 C_6_H_5_K_3_O_7_·H_2_0, 1.79 C_6_H_5_Na_3_O_7_·2H_2_0, 0.18 K_2_SO_4_, 1.32 CaCl_2_·2H_2_0, 0.65 MgCl_2_·6H_2_0, 0.30 K_2_CO_3_, and 0.60 KCl. The pH was adjusted to 6.6 with KOH. The diluted yak and cow casein micelle solutions were blended in different mass ratios, yielding mixtures containing 0% (Y0), 25% (Y25), 50% (Y50), 75% (Y75), and 100% (Y100) yak casein micelles. All mixtures were stirred at 750 rpm for 4 h at 20 °C before further analysis.

### 4.2. Particle Size of Yak–Cow Casein Micelle Complex Determination

The particle size of mixed yak and cow casein micelle solutions was determined according to Zhang [11], using a nanoparticle size and zeta potential analyzer (BT-90, Bettersize Instruments Ltd., Dandong, Liaoning, China). Before measurement, samples were diluted 100-fold with phosphate buffer (20 mM, pH 6.7) that had been pre-filtered through a 0.22-μm membrane. Approximately 1.5 mL of the diluted samples was transferred into a standard quartz cuvette and placed in the temperature-controlled sample chamber of the instrument. Measurements were performed at 25.0 °C. In the Bettersize particle size analysis software, the viscosity and refractive index of the dispersion medium (water) were set to 0.8936 cP and 1.33, respectively, while the refractive index of casein micelles was set to 1.59. Each sample was measured in triplicate.

### 4.3. Analysis of Coagulation Characteristics of Yak–Cow Casein Micelle Solutions

The pH of the mixed yak–cow casein micelle solution was adjusted to 6.1 by using lactic acid. Rennet (8901 IMCU·g−1; Caglioficio Clerici S.p.A., Eupilio, Italy) was then added at a final concentration of 1.2 g·L^−1^, and the coagulation reaction was carried out at 35 °C.

#### 4.3.1. κ-Casein Hydrolysis

Immediately after rennet addition, timing was initiated. κ-Casein hydrolysis was conducted at 35 °C and terminated at 0, 1, 2, 3, 5, 8, 12, 20, and 60 min with the addition of trichloroacetic acid (TCA) to a final concentration of 200 mg·mL^−1^, following the method of Panthi [34]. The samples were mixed and centrifuged at 6850× *g* for 30 min. The resulting supernatants were filtered through a 0.22-μm membrane, and a 100-μL aliquot was subjected to high-performance liquid chromatography (HPLC; Agilent 1100, Agilent Technologies, Santa Clara, CA, USA). Separation was performed using a C18 column (250 × 4.6 mm, 5 µm particle size) maintained at 35 °C, with UV detection at 226 nm. The mobile phase consisted of ultrapure water containing 0.1% (*v*/*v*) trifluoroacetic acid (TFA; phase A) and acetonitrile solution containing 0.1% (*v*/*v*) TFA (phase B). Due to the lack of glycomacropeptide (GMP) standards from yak and cow casein micelles, the maximum GMP level obtained after 60 min of hydrolysis was defined as 100%. GMP release kinetics were expressed as the ratio of the GMP peak area at each time point to the corresponding peak area at 60 min for yak or cow casein micelles.

#### 4.3.2. Rheological Properties

The rheological properties of mixed yak–cow casein micelle solutions during enzymatic gelation were measured using a rotational rheometer (MCR 92, Anton Paar, Graz, Austria), following the method of Lorenzen [35]. A parallel-plate geometry (60 mm diameter) was employed, and a solvent trap was used to minimize moisture evaporation throughout the experiment. Specifically, preheated samples were loaded onto the center of the lower plate, after which the upper plate was lowered to a fixed gap of 1.0 mm and excess material at the plate edges was carefully removed. Following a 10-min temperature equilibration at 35 °C, a defined volume of rennet solution was introduced at the interface between the sample and the plate edge using a micropipette. A time-sweep test was immediately initiated under constant oscillatory strain (0.5%) and a fixed angular frequency (1 rad/s). The storage modulus (G’) was continuously monitored for 1 h, with data points recorded at 50-s intervals.

#### 4.3.3. Intermolecular Forces of Casein Gels

The intermolecular forces stabilizing the casein gel network were evaluated according to the method described by Ogbon [36]. This approach is based on the selective extractions of proteins from casein gels using different solvent systems, providing a semi-quantitative assessment of the covalent and non-covalent interactions contributing to gel structure. Briefly, 4 g of rennet-induced casein gel was transferred into a centrifuge tube, and 16 mL of one of the following extraction solutions was added: deionized water, 6 M urea, 10 g·L^−1^ sodium dodecyl sulfate (SDS), or 2 mM EDTA (pH 10.0). The mixtures were homogenized by stirring at 9000 r/min for 1 min and centrifuged at 86,000× *g* for 40 min at 20 °C. The supernatants were collected, and the total protein concentration in each extract was determined by the Lowry method, as described by Rizvi [37].

### 4.4. Microstructure of Rennet-Induced Casein Gels

#### 4.4.1. Scanning Electron Microscopy (SEM)

Rennet-induced casein gels were dehydrated by vacuum freeze-drying for 48 h. The dried samples were sputter-coated with gold using an ion sputter coater at 15 mA for 60 s to improve electrical conductivity. The coated samples were examined using a scanning electron microscope (S-3400N, Hitachi High-Technologies Corporation, Minato-ku, Tokyo, Japan). SEM observations were performed at an accelerating voltage of 15.0 kV and a working distance of approximately 10.0 mm, using a backscattered electron detector. Micrographs were captured at a magnification of 1000× [38].

#### 4.4.2. Confocal Laser Scanning Microscopy (CLSM)

Approximately 1 mL of mixed casein micelle solution was stained with 10 μL of Fast Green (1 g·L^−1^, in distilled water; Sigma-Aldrich, St. Louis, MO, USA) and gently mixed for 30 min. Rennet was then added at a final concentration of 1.2 g·L^−1^, and timing was initiated immediately. An aliquot (20 µL) of the stained sample was transferred to a chambered glass slide and covered with a glass coverslip. The microstructure of the rennet-induced casein gels after incubation at 35 °C for 40 min was observed using a confocal laser scanning microscope (LSCM 800, Carl Zeiss AG, Oberkochen, Baden-Württemberg, Germany) [39].

### 4.5. Texture Profile Analysis of Enzymatic Gels

A volume of 100 mL of mixed solution was transferred into a 100-mL beaker, and rennet was added to a final concentration of 1.2 g·L^−1^. The mixture was incubated at 35 °C for 40 min to allow enzyme-induced gel formation. Following gelation, the sample was placed on a test platform of the texture analyzer (Instron Corporation, Norwood, MA, USA). Gel strength was determined using a PA/BE-d35 cylindrical probe under the following test conditions: a test speed of 1 mm/s and a compression depth of 60% of the original sample height [40].

### 4.6. Statistical Analysis

All experiments were performed in triplicate. Statistical analyses were conducted using IBM SPSS Statistics 31.0, with a statistical significance set at *p* < 0.05. Data were analyzed by one-way analysis of variance (ANOVA) followed by Tukey’s post hoc test for multiple comparisons. Figures were generated in Origin 9.8.

## Figures and Tables

**Table 1:** Hardness, chewiness, and gumminess of rennet-induced yak–cow casein gels.

Samples	Hardness (g)	Chewiness (g)	Gumminess (g)
Y0	76.20±14.21 ^b^	9.54±2.09 ^c^	24.96±4.53 ^c^
Y25	78.63±4.38 ^b^	8.76±1.87 ^c^	23.40±2.54 ^c^
Y50	84.95±6.64 ^b^	11.97±1.89 ^bc^	28.50±3.45 ^bc^
Y75	111.63±6.16 ^a^	18.21±1.74 ^a^	39.65±2.05 ^a^
Y100	106.78±9.44 ^a^	13.47±2.55 ^b^	33.93±3.55 ^ab^

Note: Values are expressed as mean ± standard deviation (*n* = 3). Different superscript letters (a–c) within the same column represent significant differences (*p* < 0.05).

## Data Availability

The original contributions presented in this study are included in the article. Further inquiries can be directed to the corresponding author(s).
